# Association between behavioral phenotypes and sustained use of smartphones and wearable devices to remotely monitor physical activity

**DOI:** 10.1038/s41598-021-01021-y

**Published:** 2021-11-02

**Authors:** Sarah J. Fendrich, Mohan Balachandran, Mitesh S. Patel

**Affiliations:** 1 Penn Medicine Nudge Unit, University of Pennsylvania, Philadelphia, PA USA; 2 Department of Medicine, University of Pennsylvania Perelman School of Medicine, Philadelphia, PA USA; 3 Crescenz Veterans Affairs Medical Center, Philadelphia, PA USA

**Keywords:** Human behaviour, Lifestyle modification, Weight management

## Abstract

Smartphones and wearable devices can be used to remotely monitor health behaviors, but little is known about how individual characteristics influence sustained use of these devices. Leveraging data on baseline activity levels and demographic, behavioral, and psychosocial traits, we used latent class analysis to identify behavioral phenotypes among participants randomized to track physical activity using a smartphone or wearable device for 6 months following hospital discharge. Four phenotypes were identified: (1) more agreeable and conscientious; (2) more active, social, and motivated; (3) more risk-taking and less supported; and (4) less active, social, and risk-taking. We found that duration and consistency of device use differed by phenotype for wearables, but not smartphones. Additionally, “at-risk” phenotypes 3 and 4 were more likely to discontinue use of a wearable device than a smartphone, while activity monitoring in phenotypes 1 and 2 did not differ by device type. These findings could help to better target remote-monitoring interventions for hospitalized patients.

## Introduction

Despite the well-established benefits of physical activity for both physical and mental health^1–5^, approximately 50% of Americans fail to achieve the minimum recommended level of physical activity^6^. Self-monitoring of physical activity data has demonstrated promise for increasing activity levels and decreasing health risks^7^, particularly when paired with well-designed feedback and financial incentives^8–12^. Additionally, several studies have shown the utility of leveraging information on patient activity levels (e.g., step counts) to predict clinical outcomes, including hospital readmissions^13–15^.

As a result, many stakeholders are increasingly interested in using mobile devices to monitor and change health behaviors^16^. The use of mobile activity trackers, smart watches, and smartphones provide a convenient and accurate way of tracking exercise^17,18^, overcoming the burden of collecting sometimes less reliable self-reported activity data^19^. However, there remain significant barriers to motivating regular, sustained use of these tracking technologies^17,20^.

A primary challenge posed by the use of mobile tracking devices is that people tend to discontinue use over time^17,21,22^. In a recent study, members of our group randomly assigned patients discharged home from the hospital to track physical activity using either a smartphone or wearable device for 6 months^22^. In this study, 61.2% of smartphone users were still tracking physical activity at 6 months compared to 46.5% of those given a wearable device, representing a significant difference between groups.

While these findings suggest that smartphones might lead to higher sustained use overall, there may be differences for different groups of patients. In a previous study, subgroups of participants distinguished by personality traits, social support, risk-taking attitudes, and baseline physical activity levels displayed varying responses to interventions targeting changes in physical activity^23^. Moreover, adoption and use of remote activity monitoring technologies have been shown to vary not only by sociodemographic characteristics such as age, gender, and income^21,24^, but also by physical activity levels^25^ and social and behavioral traits. Personality traits are associated with adherence to and efficacy of remote monitoring physical activity interventions^26,27^. Additionally, social support and financial stability, for which credit score may serve as a proxy, have been linked to adherence to remote health monitoring systems^28^.

Though these characteristics are likely to independently influence adherence to and efficacy of remote monitoring interventions, previous research has yet to fully appreciate the dynamic ways in which they may interact to produce distinct behavioral profiles with meaningful implications for health behavior. For instance, while high levels of the personality trait neuroticism have been associated with poor health behaviors such as lower medication adherence, research suggests high levels of neuroticism may in fact promote healthier behavior when *co-occurring* with high levels of conscientiousness^27^. Thus, our objective was to employ a person-centered approach to understanding the socio-behavioral correlates of remote activity monitoring behaviors. One promising approach for identifying groups of individuals who may have similar characteristics, and thus may respond similarly to health behavior change interventions, is to construct “behavioral phenotypes” of users based on individual behaviors, preferences, and motivations^16^.

In this study, we leveraged demographic, behavioral, and physical activity data collected at baseline to identify underlying homogeneous subgroups of individuals using latent class analysis (LCA). We next examined associations between the resulting subgroups, termed ‘latent classes’ and hereafter referred to as behavioral phenotypes, and patterns of remote physical activity monitoring among patients randomized to use either a smartphone or wearable device. Specifically, we compared the duration and consistency of data transmission between device types among members of each behavioral phenotype, as well as between behavioral phenotypes within each device type in a series of survival and ANOVA analyses.

## Results

Participants (n = 442; 223 using smartphones and 219 using wearables) were mostly female (n = 285, 64.5%), with a mean (SD) age of 47.4 (13.2) (Table 1). Fifty-eight participants from the original randomized clinical trial^29^ were excluded due to missing credit score data. Rates of patient death (smartphones: 3 patients; wearables: 6 patients) and overall dropout including death (smartphones: 5 patients; wearables: 11 patients) were similar between arms.

**Table 1:** Baseline latent class indicators summarized by behavioral phenotype.

Latent class indicators	Overall sample n = 442	Phenotype 1	Phenotype 2	Phenotype 3	Phenotype 4	p-value^a^
		n = 158 (35.7%)	n = 105 (23.8%)	n = 86 (19.5%)	n = 93 (21.0%)	
	Mean (SD)	Mean (SD)	Mean (SD)	Mean (SD)	Mean (SD)	
Age	47.4 (13.2)	44.5 (11.8)	55.2 (10.7)	44.5 (12.0)	46.3 (15.4)	0.978
Male, N (%)	157 (35.5)	46 (29.1)	51 (48.6)	40 (46.5)	20 (21.5)	< 0.001*
Physical activity, MET minutes^b^	2311 (3154)	2368 (3391)	2647 (3186)	2117 (2689)	2014 (3107)	0.290
Extroversion (1–5, 5 = most extroverted)	3.5 (0.8)	3.6 (0.7)	3.7 (0.8)	3.6 (0.6)	3.0 (0.6)	< 0.001*
Agreeableness (1–5, 5 = most agreeable)	4.3 (0.6)	4.7 (0.3)	4.4 (0.5)	3.8 (0.6)	3.9 (0.6)	< 0.001*
Conscientiousness (1–5, 5 = most conscientious)	4.2 (0.6)	4.6 (0.3)	4.3 (0.5)	3.8 (0.6)	3.6 (0.5)	< 0.001*
Neuroticism (1–5, 5 = most neurotic)	2.7 (0.9)	2.4 (0.8)	2.4 (0.8)	3.1 (0.8)	3.3 (0.7)	< 0.001*
Openness (1–5, 5 = most open)	3.9 (0.6)	3.9 (0.6)	4.1 (0.6)	4.0 (0.6)	3.4 (0.5)	< 0.001*
Social Support (0–5, 5 = most support)^c^	4.1 (0.9)	4.2 (0.9)	4.4 (0.8)	3.8 (1.1)	3.9 (0.9)	< 0.001*
Health/safety risk-taking (1 to 7, 7 = extremely likely to engage in risky behavior)^d^	2.4 (1.2)	2.2 (1.0)	2.1 (1.1)	3.6 (1.4)	2.1 (0.9)	0.009*
Social risk-taking (1 to 7, 7 = extremely likely to engage in risky behavior)	4.8 (1.2)	4.7 (1.3)	5.1 (1.0)	5.7 (0.8)	3.8 (1.0)	0.003*
Credit score (300–850, 850 = excellent)	617 (118)	547 (76)	738 (77)	586 (107)	626 (113)	< 0.001*

SD, standard deviation.

^a^ANOVA tests were used for all variables aside from sex (male vs female), for which a chi-squared test was used.

^b^Derived from the International Physical Activity Questionnaire (IPAQ). MET minutes represent the amount of energy expended carrying out physical activity per week.

^c^Derived from the Medical Outcomes Survey (MOS), scaled down from 0–100 to 0–5.

^d^Derived from the Domain-Specific Risk-Taking (DOSPERT) Scale.

*p-value is significant (p < 0.05).

Considering model fit parameters, the results of likelihood ratio tests, the distribution of the sample across classes, and model interpretability, a 4-class LCA model was selected (Supplementary Table 1). This model yielded low values for Akaike information criterion (AIC) and sample size adjusted Bayesian information criterion (BIC) and high entropy. Though the 3-class model yielded slightly lower BIC, the likelihood ratio test for the 4-class model was statistically significant, indicating significantly improved model fit when using 4 classes compared to 3 classes. Additionally, while the 5-class model yielded slightly lower AIC and higher entropy than the 4-class model, the likelihood ratio test was not statistically significant, indicating that these differences do not significantly improve model fit. The sample was also the most evenly distributed across classes in the 4-class model.

## Behavioral phenotype descriptions

The four behavioral phenotypes differed significantly on all latent class indicators aside from age and physical activity (p-values for ANOVA test < 0.01; Table 1). Phenotypes also differed significantly on a number of additional sociodemographic criteria such as race, insurance type, education level, marital status, and household income (Table 2). However, they did not differ in body mass index or Charlson comorbidity index.

**Table 2:** Baseline sociodemographic characteristics included in cox proportional hazard models, summarized by behavioral phenotype.

Variable	Phenotype 1	Phenotype 2	Phenotype 3	Phenotype 4	p-value^a^
	n = 158 (35.7%)	n = 105 (23.8%)	n = 86 (19.5%)	n = 93 (21.0%)	
	n (%)	n (%)	n (%)	n (%)	
**Study arm**					0.014*
Smartphone	82 (51.9)	53 (50.5)	53 (61.6)	35 (37.6)	
Wearable	76 (48.1)	52 (49.5)	33 (38.4)	58 (62.4)	
**Gender**					< 0.001*
Male	46 (29.1)	51 (48.6)	40 (46.5)	20 (21.5)	
Female	112 (70.9)	54 (51.4)	46 (53.5)	73 (78.5)	
**Race**					< 0.001*
Non-Hispanic White	35 (22.2)	76 (72.4)	40 (46.5)	54 (58.1)	
Non-Hispanic Black	106 (67.1)	19 (18.1)	36 (41.9)	31 (33.3)	
Hispanic	8 (5.1)	6 (5.7)	5 (5.8)	5 (5.4)	
Other	9 (5.7)	4 (3.8)	5 (5.8)	3 (3.2)	
**Insurance type**					< 0.001*
Commercial	66 (41.8)	70 (66.7)	46 (53.5)	50 (53.8)	
Medicare	55 (34.8)	33 (31.4)	22 (25.6)	32 (34.4)	
Medicaid	36 (22.8)	2 (1.9)	17 (19.8)	11 (11.8)	
**Education level**					< 0.001*
Less than high school	16 (10.1)	2 (1.9)	7 (8.1)	5 (5.4)	
High school graduate	100 (63.3)	45 (42.9)	52 (60.5)	62 (66.7)	
College graduate	42 (26.6)	58 (55.2)	27 (31.4)	26 (28.0)	
**Marital status**					< 0.001*
Single, never married	83 (52.5)	19 (18.1)	45 (52.3)	38 (40.9)	
Married or domestic partnership	30 (19.0)	10 (9.5)	16 (18.6)	21 (22.6)	
Other	45 (28.5)	76 (72.4)	25 (29.1)	34 (36.6)	
**Household income**					< 0.001*
< 50,000	67 (42.4)	18 (17.1)	33 (38.4)	26 (28.0)	
50,000–100,000	24 (15.2)	27 (25.7)	15 (17.4)	11 (11.8)	
> 100,000	7 (4.4)	35 (33.3)	15 (17.4)	14 (15.1)	
Declined to respond	60 (38.0)	25 (23.8)	23 (26.7)	42 (45.2)	
Age, mean (SD)	44.5 (11.8)	55.2 (10.7)	44.5 (12.0)	46.3 (15.4)	0.969
Body mass index, mean (SD)^b^	31.9 (9.1)	29.3 (7.1)	31.9 (9.3)	30.3 (9.9)	0.550
CCI score, median (IQR)	4 (4)	3 (4)	3 (4)	3 (4)	0.370

SD, standard deviation; CCI, Charlson Comorbidity Index; IQR, interquartile range.

^a^Chi-Squared test, aside from age, BMI, and CCI, for which one-way ANOVA tests were used. *p-value is significant (p < 0.05).

^b^Calculated as weight in kilograms divided by height in meters squared.

**Figure 1:**
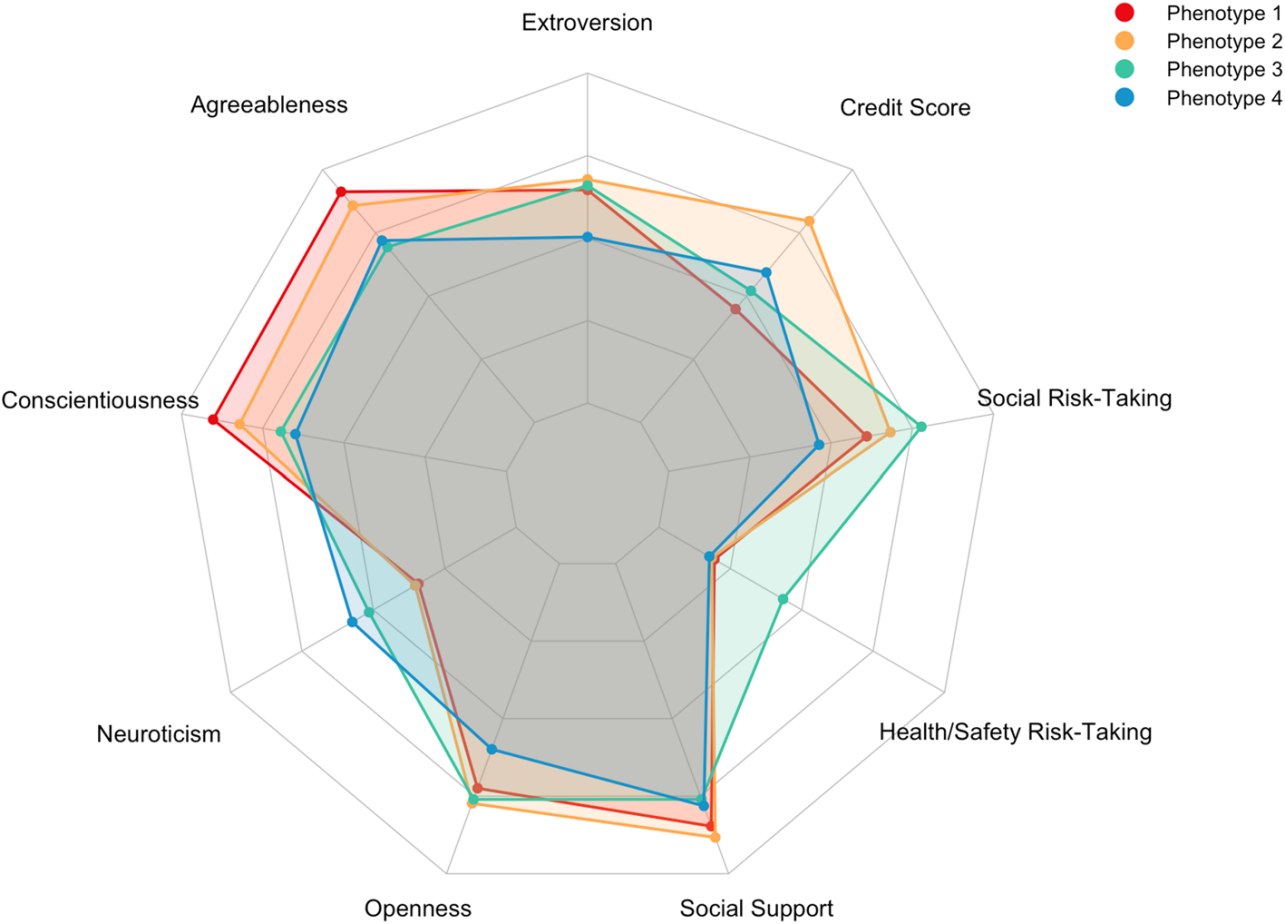
Radar chart comparing behavioral profiles. Points reflect mean scores for each group on the latent class indicators coded as continuous variables. Axis boundaries are the minimum and maximum possible values for each measure. Age, gender, and physical activity were excluded.

Phenotypes were characterized by the key latent class indicators driving group distinctions (Fig. 1; Table 3). Below we elaborate on these defining features and on sociodemographic characteristics of note.

**Table 3:** Key factors driving behavioral phenotype (i.e., latent class) distinctions.

Phenotype 1 More agreeable and conscientious n = 158, 35.7%	Phenotype 2 More active, social, and motivated n = 105, 23.8%	Phenotype 3 More risk-taking, less supported n = 86, 19.5%	Phenotype 4 Less active, social, and risk-taking n = 93, 21.0%
Higher agreeableness	Higher physical activity	Lower agreeableness	Lower physical activity
Higher conscientiousness	Higher openness	Lower social support	Lower extroversion
Lower neuroticism	Higher extroversion	Higher health safety risk taking	Lower conscientiousness
Lower credit score	Higher social support	Higher social risk taking	Higher neuroticism
	Older		Lower openness
	Higher credit score		Lower health safety risk taking
			Lower social risk taking
			Fewer males

### Phenotype 1—more agreeable and conscientious

Phenotype 1 was the largest subgroup, comprising 35.7% of the sample (n = 158). This phenotype scored the highest in agreeableness (+ 0.67 SD above the sample mean) and conscientiousness (+ 0.67 SD), with participants in this phenotype only reporting high levels of both features (Supplementary Table 2). They also had the lowest credit scores (− 0.59 SD) and are characterized by lower scores in neuroticism (− 0.33 SD). In terms of sociodemographic features, phenotype 1 was composed of more non-Hispanic Black participants and participants with lower income and levels of education.

### Phenotype 2—more active, social, and motivated

Phenotype 2 comprised 23.8% of the sample (n = 105). This phenotype was older than the overall sample (+ 0.59 SD from sample mean) and males were overrepresented in this phenotype relative to the whole sample. Participants in this phenotype reported the highest number of MET minutes per week across classes (+ 0.11 SD), which reflect the amount of energy expended carrying out physical activity. They also scored the highest in openness (+ 0.33 SD), extroversion (+ 0.25 SD), and social support (+ 0.33 SD), and had the highest credit scores (+ 1.03 SD), reflecting a greater degree of social and motivated behavior. There were fewer non-Hispanic Black participants and more Hispanic participants, college graduates, and higher income participants in this phenotype relative to the overall sample.

### Phenotype 3—more risk-taking, less supported

Phenotype 3 comprised 19.5% of the sample (n = 86). This phenotype reported the highest levels of risk-taking in both the health and safety domain (+ 1.00 SD) and social domain (+ 0.75 SD). Other defining characteristics include the lowest scores in social support (− 0.33 SD) and agreeableness (− 0.83 SD). Males were also overrepresented in this phenotype relative to the whole sample, as were participants on Medicaid.

### Phenotype 4—less active, social, and risk-taking

Phenotype 4 comprised 21% of the sample (n = 93). Participants in this phenotype reported the lowest levels of physical activity (− 0.09 SD) and social risk-taking preferences (− 0.83 SD). They also scored the lowest in extroversion (− 0.63 SD), conscientiousness (− 1.00 SD), and openness (− 0.83 SD), and the highest in neuroticism (+ 0.66 SD). Males were underrepresented in this group.

### Behavioral phenotype and sustained device use

#### Differences within each phenotype

**Figure 2:**
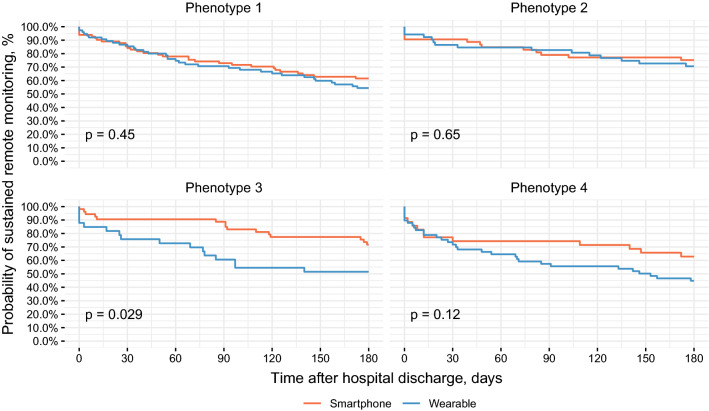
Kaplan–Meier survival plots displaying duration of sustained remote monitoring of physical activity data after hospital discharge to home across device types, stratified by behavioral phenotype. P-values are from unadjusted log-rank tests.

Figure 2 shows the proportion of participants in each phenotype providing data over the 180 days after hospital discharge, comparing patients randomized to use smartphones versus wearable devices. The more agreeable and conscientious phenotype 1 and more active, social, and motivated phenotype 2 showed no differences in duration of data provision with smartphones versus wearables. However, the more risk-taking and less supported phenotype 3 and less active, social, and risk-taking phenotype 4 showed an increased likelihood to discontinue use of a wearable compared to a smartphone, though this difference was only statistically significant in phenotype 3 (unadjusted log rank test: p = 0.029).

The increased likelihood to discontinue wearable use in phenotype 3 remains significant in cox proportional hazard models adjusted for sociodemographic characteristics (Table 4). Consistent with the unadjusted analyses, smartphones and wearable devices did not differ significantly in the other phenotypes. Results do not change appreciably in a sensitivity analysis defining a day of data transmission as a day with at least 1000 steps reported (Supplementary Table 3).

**Table 4:** Cox proportional hazard models associating study arm with last day of data transmission, censoring on patient death and adjusting for patient-level sociodemographic characteristics.

Variable	Phenotype 1		Phenotype 2		Phenotype 3		Phenotype 4	
	n = 158, n events = 65 (41.1%)		n = 105, events = 28 (26.7%)		n = 86, events = 31 (36.0%)		n = 93, events = 44 (47.3%)	
	HR (95% CI)	p-value	HR (95% CI)	p-value	HR (95% CI)	p-value	HR (95% CI)	p-value
**Study arm**								
Smartphone	Ref.		Ref.		Ref.		Ref.	
Wearable	1.14 (0.67–1.95)	0.622	1.24 (0.53–2.91)	0.619	4.36 (1.68–11.37)	0.003*	1.50 (0.73–3.08)	0.269
Age	1.00 (0.98–1.03)	0.813	0.98 (0.93–1.03)	0.334	1.01 (0.97–1.05)	0.668	1.02 (0.98–1.05)	0.354
**Gender**								
Male	Ref.		Ref.		Ref.		Ref.	
Female	1.15 (0.62–2.13)	0.652	1.66 (0.68–4.03)	0.264	1.21 (0.50–2.92)	0.672	1.39 (0.59–3.30)	0.456
**Race**								
Hispanic	Ref.		Ref.		Ref.		Ref.	
Non-Hispanic Black	1.15 (0.36–3.66)	0.816	0.19 (0.02–1.51)	0.116	1.56 (0.15–16.00)	0.707	1.23 (0.24–6.30)	0.806
Non-Hispanic White	0.44 (0.12–1.61)	0.214	0.69 (0.15–3.26)	0.644	0.98 (0.10–9.86)	0.987	2.09 (0.43–10.26)	0.362
Other	1.31 (0.31–5.48)	0.714	0.35 (0.03–4.70)	0.430	0.72 (0.04–13.10)	0.824	2.51 (0.30–20.64)	0.393
**Insurance type**								
Commercial	Ref.		Ref.		Ref.		Ref.	
Medicare	0.75 (0.38–1.48)	0.408	0.93 (0.30–2.87)	0.903	3.29 (1.06–10.23)	0.040*	0.91 (0.39–2.15)	0.836
Medicaid	1.15 (0.58–2.29)	0.683	8.06 (0.64–101.37)	0.106	5.60 (1.57–19.92)	0.008*	2.31 (0.79–6.76)	0.128
**Education level**								
Less than high school	Ref.		Ref.		Ref.		Ref.	
High school graduate	0.67 (0.30–1.47)	0.312	0.45 (0.04–4.44)	0.491	4.60 (0.87–24.23)	0.072	1.45 (0.30–6.90)	0.642
College graduate	0.58 (0.23–1.44)	0.243	0.26 (0.03–2.58)	0.247	8.72 (1.12–67.58)	0.038*	0.76 (0.13–4.49)	0.763
**Marital status**								
Single, never married	Ref.		Ref.		Ref.		Ref.	
Married or domestic partnership	0.97 (0.49–1.91)	0.931	1.53 (0.33–7.08)	0.584	0.44 (0.14–1.43)	0.173	1.13 (0.47–2.73)	0.784
Other	1.82 (0.82–4.02)	0.141	2.35 (0.39–14.06)	0.348	0.53 (0.13–2.16)	0.372	0.82 (0.27–2.49)	0.724
**Household income**								
< 50,000	Ref.		Ref.		Ref.		Ref.	
50,000–100,000	0.67 (0.26–1.72)	0.407	0.54 (0.11–2.56)	0.437	1.93 (0.59–6.34)	0.281	0.69 (0.22–2.20)	0.534
> 100,000	2.43 (0.64–9.28)	0.194	1.11 (0.20–6.18)	0.905	3.10 (0.63–15.23)	0.164	0.23 (0.05–1.01)	0.051
Declined to respond	1.08 (0.61–1.90)	0.791	0.79 (0.16–3.82)	0.768	1.16 (0.39–3.44)	0.784	0.73 (0.33–1.59)	0.423
BMI	0.99 (0.96–1.02)	0.367	1.01 (0.96–1.07)	0.652	0.96 (0.92–1.00)	0.071	1.01 (0.98–1.05)	0.508
CCI	0.98 (0.89–1.09)	0.770	1.19 (1.02–1.39)	0.025*	1.00 (0.87–1.15)	0.977	0.89 (0.76–1.04)	0.140

Models were fit separately for each behavioral phenotype.

HR, hazard ratio; CI, confidence interval; BMI, body mass index; CCI, Charlson Comorbidity Index.

*p-value is significant (p < 0.05).

ANOVA analyses comparing the proportion of days with step data provided during the 180-day study period reveal significantly more consistent activity monitoring in smartphone users compared to wearable users in both the more risk-taking and less supported phenotype 3 and less active, social, and risk-taking phenotype 4 (p = 0.014 and p = 0.047, respectively; Supplementary Table 4). In the sensitivity analysis these differences are not statistically significant, though the increased consistency in data provision among smartphone users relative to wearable users shows trend level significance in phenotype 3 (p = 0.063; Supplementary Table 4).

#### Differences between phenotypes

**Figure 3:**
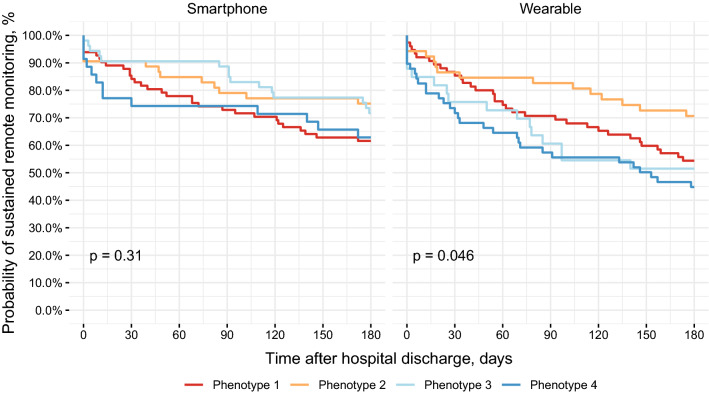
Kaplan–Meier survival plots displaying duration of sustained remote monitoring of physical activity data after hospital discharge to home across behavioral phenotypes, stratified by device type. P-values are from unadjusted log-rank tests. Across both device types there is an initial drop-off in the number of participants providing data in the less active, social, and risk-taking phenotype 4: within the first week, 20.7% of wearable users and 14.3% of smartphone users stopped providing data. In the more risk-taking and less supported phenotype 3, we observe similarly high rates of initial drop-off only in the wearable arm, with 15.2% of wearable users discontinuing use within the first week, compared to only 5.7% in the smartphone arm. Rates of first-week drop-off in phenotypes 1 and 2 were lower and relatively consistent between arms, ranging from approximately 5–9%.

Unadjusted log rank tests show a significant difference in data provision between phenotypes among wearable users (p = 0.046), but not among smartphone users (p > 0.05; Fig. 3). In adjusted cox proportional hazard models, the log-rank test comparing phenotypes among wearable users trends towards significance (p = 0.051), as does the decreased likelihood to stop providing data in the more agreeable and conscientious phenotype 1 and more active, social, and motivated phenotype 2 relative to the less active, social, and risk-taking phenotype 4 (Table 5). Findings were similar in the sensitivity analysis (Supplementary Table 5).

**Table 5:** Cox proportional hazard models associating behavioral phenotype with last day of data transmission, censoring on patient death and adjusting for patient-level sociodemographic characteristics.

Variable	Smartphone		Wearable	
	n = 223, n events = 72 (32.3%)		n = 219, n events = 96 (43.8%)	
	HR (95% CI)	p-value	HR (95% CI)	p-value
**Behavioral phenotype**				
Phenotype 1	0.94 (0.47–1.90)	0.865	0.59 (0.34–1.02)	0.060
Phenotype 2	0.75 (0.32–1.79)	0.523	0.55 (0.28–1.09)	0.086
Phenotype 3	0.73 (0.33–1.60)	0.437	0.74 (0.38–1.44)	0.368
Phenotype 4	Ref.		Ref.	
Age	1.01 (0.98–1.03)	0.552	0.99 (0.97–1.01)	0.478
**Gender**				
Male	Ref.		Ref.	
Female	1.39 (0.80–2.40)	0.240	1.20 (0.75–1.94)	0.451
**Race**				
Hispanic	Ref.		Ref.	
Non-Hispanic Black	1.28 (0.39–4.21)	0.685	0.53 (0.21–1.34)	0.178
Non-Hispanic White	1.01 (0.32–3.21)	0.992	0.58 (0.23–1.47)	0.247
Other	1.08 (0.24–4.83)	0.918	0.75 (0.23–2.48)	0.640
**Insurance type**				
Commercial	Ref.		Ref.	
Medicare	0.77 (0.43–1.40)	0.394	1.20 (0.67–2.14)	0.542
Medicaid	1.37 (0.67–2.81)	0.393	1.89 (0.97–3.68)	0.062
**Education level**				
Less than high school	Ref.		Ref.	
High school graduate	1.57 (0.52–4.73)	0.423	0.72 (0.36–1.45)	0.354
College graduate	0.87 (0.26–2.92)	0.822	0.73 (0.34–1.61)	0.440
**Marital status**				
Single, never married	Ref.		Ref.	
Married or domestic partnership	0.86 (0.44–1.69)	0.671	0.94 (0.53–1.66)	0.832
Other	1.20 (0.55–2.60)	0.645	1.06 (0.54–2.07)	0.867
**Household income**				
< 50,000	Ref.		Ref.	
50,000–100,000	1.31 (0.61–2.84)	0.489	0.64 (0.31–1.31)	0.220
> 100,000	1.74 (0.69–4.41)	0.242	0.59 (0.25–1.38)	0.224
Declined to respond	1.42 (0.77–2.62)	0.263	0.71 (0.43–1.18)	0.187
BMI	0.97 (0.94–1.00)	0.051	1.00 (0.98–1.03)	0.800
CCI	1.04 (0.96–1.14)	0.336	1.00 (0.92–1.08)	0.973

Models were fit separately for each study arm.

HR, hazard ratio; CI, confidence interval; BMI, body mass index; CI, Charlson Comorbidity Index; Ref, reference level.

There were no significant differences between phenotypes in the proportion of days with data provided in either device type (Supplementary Table 6). However, there are trend level differences between phenotypes in both device types in the sensitivity analysis (smartphones: p = 0.057, wearables: p = 0.093; Supplementary Table 6). Otherwise, results did not change appreciably in the sensitivity analysis (Supplementary Table 6).

## Discussion

In this study, we demonstrate that “behavioral phenotypes,” or subgroups of individuals defined by co-occurring social, behavioral, psychological, and demographic traits had different patterns of sustained use of smartphones and wearable devices for tracking physical activity. In prior work, the overall sample had higher sustained use in the smartphone group when compared to the wearable group^22^. Our findings demonstrate that this only holds true for a subset of “at-risk” individuals. Specifically, duration and consistency of sustained activity monitoring differed by device type only in the more risk-taking and less supported phenotype 3 and less active, social, and risk-taking phenotype 4. In both phenotypes, smartphone users displayed significantly greater tracking consistency compared to wearable users, while duration of activity monitoring was only statistically significantly higher among smartphone users in the more risk-taking and less supported phenotype 3. Additionally, behavioral phenotypes differed in duration of activity monitoring *only* among wearable users, not among smartphone users. This is driven in part by the high rates of early drop-off in use of wearable devices observed in the more risk-taking and less supported phenotype 3 and less active, social, and risk-taking phenotype 4.

The less sustained activity monitoring seen in these phenotypes is in line with previous research. The more risk-taking and less supported phenotype 3 and less active, social, and risk-taking phenotype 4 reported the lowest baseline physical activity levels, which research has linked to a decreased likelihood of forming successful activity tracking habits^30^. These phenotypes also both display personality traits seen in “Type D” or distressed personality type, a well-established phenotype marked by a tendency toward negative emotions and social inhibition. Type D personality is correlated with high levels of neuroticism and low levels of conscientiousness, agreeableness, and social support, seen in both phenotypes 3 and 4, and has been associated with poor health outcomes and a sedentary lifestyle^31–33^.

Whereas the less active, social, and risk-taking phenotype 4 displayed poor activity monitoring performance across both device types, the more risk-taking and less supported phenotype 3 was a top performer among smartphone users, showing less sustained and consistent activity monitoring only among wearable users. This may be because the more risk-taking and less supported phenotype 3 diverges from the less active, social, and risk-taking phenotype 4 and the Type D profile on a number of traits related to sociability: phenotype 3 displays high extraversion, high openness, and high degrees of social risk taking, while phenotype 4 reported the lowest scores on these metrics. This suggests that sustained use of wearable devices may be more reliant on characteristics such as neuroticism, conscientiousness, and social support, while sustained activity monitoring using smartphone devices may be more dependent on traits related to sociability and openness. Future research might seek to investigate other baseline characteristics that may mediate the relationship between these characteristics and successful physical activity monitoring via a smartphone device, such as general daily smartphone use.

The more active, social, and motivated phenotype 2 displayed high levels of sustained and consistent activity monitoring across device types, most notably showing increased adherence to activity monitoring compared to the other phenotypes in the wearable arm. Though this is generally unsurprising, a driving feature of this phenotype was older age, which contrasts with previous research suggesting older adults are less likely to develop successful habits using wearable activity monitoring technology^34,35^. It is possible that the high levels of social support reported by this phenotype, in addition to support from the study team, may have partially offset initial acceptance and usability barriers oft-cited in older adults^35,36^. This reflects the importance of considering individual differences when examining patterns in and barriers to use of monitoring technologies in older adults, particularly given that they represent a rapidly growing segment of the population likely to benefit from activity monitoring technologies^34^.

In this study we directly compare activity monitoring use patterns between device types while accounting for differences in users’ behavioral, psychological, and demographic profiles. Findings suggest that smartphones may be a better option when prioritizing the scalability of activity monitoring interventions, given that smartphone users provided at least as many days of step data as wearable users across all four behavioral phenotypes. However, granted that wearables did not underperform smartphones among all subgroups of individuals, trade-offs between device types should be considered within the context of the goals and target population of a specific program. For instance, while most people already own a smartphone^37^, which reduces program costs and barriers related to users forgetting to carry or charge an extra device^38^, not everyone carries their phone on them throughout the entire day, so some activity may not be recorded. Additionally, wearables can track biometric and sleep data that smartphones cannot^29^. When feasible, we recommend conducting qualitative assessments of user characteristics as well as preferences and perceived barriers to adoption and use to better tailor remote-monitoring interventions to the given population.

## Limitations

This study is limited in that it is a secondary analysis of a randomized control trial, which was not designed to detect differences across two study arms and four subgroups of participants. Thus, the analyses in the current study may be underpowered. Additionally, our sample consists of patients within one health system and who had recently been discharged from the hospital, which may limit the generalizability of our findings. Nevertheless, the divergence in activity monitoring patterns we identify within subgroups of this sample points to the utility of addressing individual variation in traits related to health behaviors and technology use.

## Conclusion

To our knowledge, our study is the first to investigate the association between socio-behavioral profiles and sustained physical activity tracking, notably comparing monitoring patterns across multiple device types. We demonstrate the importance of accounting for individual differences in the implementation and evaluation of activity monitoring programs. Four “behavioral phenotypes” of participants differentiated by personality traits, behavioral tendencies, and social resources showed distinct patterns in the sustained duration and consistency of remote activity monitoring, particularly among individuals randomized to use wearable devices. We find that “at-risk” phenotypes characterized by tendencies toward negative affect and lower levels of baseline physical activity and social support were more likely to discontinue use of wearable devices.

The differences in adherence to wearable- versus smartphone-mediated activity tracking we identify across behavioral subgroups point to the presence of distinct barriers to activity tracking experienced by different populations. Future research should aim to establish socio-behavioral profiles in larger populations and characterize the unique barriers associated with them—particularly among potentially at-risk profiles as described here—to inform the strategic design of remote-monitoring technologies and the health-promoting interventions reliant on them.

## Methods

### Study design

This is a secondary analysis of a randomized clinical trial (ClinicalTrials.gov identifier: NCT02983812). The design and protocol of the trial have been previously published^29^. Patients were approached in-hospital between January 23, 2017 to January 7, 2019 and were eligible for participation in the trial if they were above the age of 18, could ambulate, had a smartphone compatible with the Withings HealthMate application, and planned to be discharged to home.

Prior to hospital discharge, patients were randomly assigned to track their physical activity for 6 months using a smartphone or a wrist-worn wearable device. Participants in the smartphone arm tracked their physical activity using the Withings HealthMate application, connected via any compatible smartphone device. Participants in the wearable arm used a Withings Steel device provided by the study team with a battery lasting approximately 8 months.

All participants received $50 to enroll and $50 upon trial completion. To level incentives across study arms, participants assigned to use a smartphone alone were also given the wearable device after completing the trial. For each participant, the first day of the 6-month study began day one after they were discharged. This study was approved by the University of Pennsylvania Institutional Review Board and participants provided written informed consent to participate in the clinical trial. All methods were performed in accordance with the relevant guidelines and regulations.

Using the Withings HealthMate application, physical activity data were transmitted from the devices to Way to Health^39^, a research technology platform used in prior work for activity interventions involving remote monitoring^8,9,40–44^. If data had not been transmitted for four consecutive days, patients were sent a notification to synchronize their device via their selected communication preference (text message, email, or telephone voice recording). A day of data transmission was defined as a day in which more than zero steps were reported.

### Latent class analysis variable selection

During enrollment, participants were asked to complete a sociodemographic survey and series of validated instruments to evaluate physical activity level^45^, personality^46^, risk-taking preferences^47^, and social support^48^. Data on patient credit scores (VantageScore V3) were obtained from Experian within 6 months of hospital discharge.

Variables were selected for inclusion in LCA model construction based on established associations with physical activity intervention responsiveness and success of remote health monitoring^21,23–28^. Indicators with insufficient variability were excluded given that they are unlikely to aid in identifying subgroups. All variables were converted into categorical variables in order to be included in the LCA, which requires discrete input^49^.

Latent class indicators across the following domains were included:

- *Demographics*, including age (coded as 18–34 years, 35–49 years, and > 50 years) and sex.
- *Baseline physical activity*, which was assessed using the International Physical Activity Questionnaire and scored as low, moderate, and high levels^50^.
- *Risk-taking preferences* were assessed based on patients’ self-reported likelihood to engage in risky behaviors related to health/safety and social situations, measured using the DOSPERT survey. The DOSPERT uses a 7-point Likert scale and was converted into low (1–2.9), medium (3–4.9), and high (5–7) levels.
- *Social support* was measured using the overall score on the Medical Outcomes Study (MOS) Social Support survey, computed as the average of subscores assessing emotional/informational support, tangible support, affectionate support, and positive social interactions.
- *Personality* was assessed using the Big Five traits of extroversion, agreeableness, conscientiousness, neuroticism, and openness. The MOS and Big Five surveys both use 5-point Likert scales, and were converted to low (1–2.9), medium (3–3.9), and high (4–5) levels of each trait, as done in previous work^23^.

### Statistical analyses

LCA is a statistical method used to identify distinct subgroups within a population based on patterns discerned among at least two observed dependent variables (‘latent class indicators’)^51^. Given a set of latent class indicators, the objective of LCA is to determine the optimal number of subgroups, or ‘latent classes’, to divide a population into such that latent classes are sufficiently distinct, and individuals can be categorized into their most likely class with high accuracy.

LCA was selected because it has demonstrated superior performance to other common classification techniques such as multidimensional scaling and cluster analysis in its reliability and accuracy, ability to objectively evaluate model fit, and balance of parsimony and complexity in its output^52^. This approach has previously been used to identify subgroups that differ meaningfully in response to a behavioral intervention to increase physical activity^23^ and in adherence to therapeutic interventions^53,54^.

The LCA was performed in Mplus (Version 8.2), a software package commonly used for LCA^55^. To identify the number of latent classes that yielded optimal model fit, we fit a series of latent class models beginning with the most parsimonious 2-class model and iteratively increasing the number of subgroup divisions up to five. Model fit was evaluated holistically based on quantitative measures of model fit as well as qualitative assessments of model interpretability^49^. Statistical indices of model fit considered include Akaike information criterion and Bayesian information criterion values, which are measures of prediction error, and entropy, which is a measure of classification accuracy, with higher values reflecting increased class distinctiveness^56,57^. We used the Vuong-Lo-Mendell-Rubin likelihood ratio test (LRT) to evaluate if adding another class statistically significantly improved model fit^57,58^. The distribution of patients throughout latent classes was also considered to maximize statistical power and model interpretability.

After determining the best fit model, we used descriptive statistics to evaluate differences in baseline and sociodemographic variables between latent classes in R (Version 3.5.1; R Foundation for Statistical Computing). Next, we characterized the key factors driving class distinctions based on group differences described in Table 1. This selection of driving factors was generally supported by an assessment of the characteristics that were either over- or underrepresented in each group. These characteristics were identified by examining the distribution of patients in each variable level (e.g., low, medium, or high degree of openness) in each class relative to the distribution in the overall sample (Supplementary Table 2). To do so, probability weights reflecting the estimated proportion of each class that fell into each level category were generated in Mplus.

To examine differences in duration of data activity monitoring between classes and study arms, we first generated survival curves using Kaplan–Meier estimates, plotting the proportion of patients providing data over the 180 days after discharge, censoring on patient death. The duration of data transmission was estimated using the last day a step value was received. Using log rank tests, we examined the unadjusted differences between study arms in each latent class, as well as between latent classes in each study arm. Subsequently, Cox proportional hazard models were fit and adjusted for age, gender, race/ethnicity, insurance, education, marital status, annual household income, body mass index, and Charlson Comorbidity Index score.

To evaluate differences in the consistency of data transmission, we compared the proportion of days of data transmission using one-way ANOVA tests. This is in line with previous research that has defined consistency of activity tracking as the percentage of days tracked relative to the number of days during a trial^59^. As a sensitivity analysis, we repeated all analyses defining a day of data transmission as a day with over 1000 steps reported, since values less than 1,000 are unlikely to capture actual activity throughout a whole day, indicating a degree of data missingness^60,61^. Investigators and analysts were blinded to group assignment.

## Supplementary Information

Supplementary Information.

## Data Availability

Ms. Fendrich had full access to all the data in the study and takes responsibility for the integrity of the data and the accuracy of the data analysis.
