# Supplementary material for: Association between behavioral phenotypes and sustained use of smartphones and wearable devices to remotely monitor physical activity
Source: Sci Rep. 2021 Nov 2;11:21501. doi: 10.1038/s41598-021-01021-y (PMC8563736; doi:10.1038/s41598-021-01021-y)
Supplement: Supplementary file 1 — Supplementary Information. [file 41598_2021_1021_MOESM1_ESM.pdf]

Supplementary Table 1. Fit statistics and number of individuals per class for latent class models with two to five classes.

Number of classes	*AIC*	*BIC* [a]	*Entropy* [b]	N_1	N_2	N_3	N_4	N_5	*p-value* [c]
2	9176.9	9220.0	0.696	329	113				0.057
3	9117.9	9183.0	0.651	245	102	95			0.482
4	9098.7	9185.9	0.716	158	105	86	93		0.033*
5	9098.2	9208.4	0.804	46	70	162	118	46	0.687

Abbreviations: AIC, Akaike information criterion; BIC, Bayesian information criterion.

[a] Adjusted for sample size

[b] Entropy is a measure of class separation with higher values indicating better separation.

[c] By the Vuong-Lo-Mendell-Rubin likelihood ratio test, testing whether increasing the number of classes provides improved model fit compared to a model using one fewer class. * p-value is significant ($p < 0.05$)

Supplementary Table 2. Relative abundance of each latent class indicator in each phenotype relative to the whole sample

	All<br>n = 442	Phenotype 1<br>n = 158, 35.7%	Phenotype 2<br>n = 105, 23.8%	Phenotype 3<br>n = 86, 19.5%	Phenotype 4<br>n = 93, 21.0%
Variable	*n (%)*	*Rel.*	*Rel.*	*Rel.*	*Rel.*
Age					
18-34 years	227 (51.4)	1.10	**0.56**	1.19	1.10
35-49 years	177 (40.0)	1.03	**1.33**	0.93	0.72
50 or greater years	38 (8.6)	**0.31**	**2.13**	**0.15**	**1.73**
Gender					
Male	157 (35.5)	0.84	**1.31**	**1.35**	**0.59**
Female	285 (64.5)	1.09	0.83	0.80	1.22
Physical Activity					
Low (1-2.9)	191 (43.2)	0.98	0.82	0.89	**1.31**
Medium (3-3.9)	131 (29.6)	1.10	1.09	1.09	**0.69**
High (4-5)	120 (27.1)	0.93	1.20	1.08	0.85
Extroversion					
Low (1-2.9)	87 (19.7)	0.77	0.96	**0.40**	**1.95**
Medium (3-3.9)	231 (52.3)	0.88	0.81	1.25	1.11
High (4-5)	124 (28.1)	**1.38**	**1.37**	0.95	**0.13**
Agreeableness					
Low (1-2.9)	18 (4.1)	**0.00**	**0.00**	**2.34**	**2.10**
Medium (3-3.9)	102 (23.1)	**0.00**	0.75	**2.16**	**1.57**
High (4-5)	322 (72.9)	**1.37**	1.13	**0.55**	0.76
Conscientiousness					
Low (1-2.9)	18 (4.1)	**0.00**	**0.29**	**2.15**	**2.00**
Medium (3-3.9)	136 (30.8)	**0.00**	**0.69**	**1.56**	**2.21**
High (4-5)	288 (65.2)	**1.53**	1.19	**0.66**	**0.36**
Neuroticism					
Low (1-2.9)	261 (59.0)	1.25	1.23	0.83	0.58
Medium (3-3.9)	142 (32.1)	**0.67**	0.76	1.21	**1.52**
High (4-5)	39 (8.8)	**0.55**	**0.34**	**1.38**	**1.94**
Openness					
Low (1-2.9)	35 (7.9)	0.91	**0.28**	**0.63**	**2.20**
Medium (3-3.9)	213 (48.2)	0.92	0.84	0.73	**1.54**
High (4-5)	194 (43.9)	1.11	**1.31**	**1.36**	**0.19**
Social Support					
Low (1-2.9)	58 (13.1)	0.74	**0.66**	**1.81**	0.93
Medium (3-3.9)	100 (22.6)	0.84	**0.52**	0.88	**1.82**
High (4-5)	284 (64.3)	1.11	1.24	0.88	0.73
Health/Safety Risk Taking					
Low (1-2.9)	306 (69.2)	1.08	1.22	**0.48**	1.17
Medium (3-4.9)	115 (26.0)	0.96	**0.49**	**1.85**	0.73
High (5-7)	21 (4.8)	**0.00**	**0.58**	**3.90**	**0.00**
Social Risk-Taking					
Low (1-2.9)	37 (8.4)	1.12	**0.00**	**0.00**	**2.75**
Medium (3-4.9)	189 (42.8)	1.09	1.01	**0.37**	**1.46**
High (5-7)	216 (48.9)	0.90	1.16	**1.72**	**0.29**
Credit Score					
300 < 600	237 (53.6)	**1.52**	**0.00**	1.29	0.93
600 - 780	143 (32.4)	**0.57**	**1.73**	**0.62**	1.27
780 - 850	62 (14.0)	**0.00**	**3.13**	0.76	**0.64**

Abbreviations: Rel, abundance relative to entire sample. Values in bold indicate a relative abundance greater than or equal to 1.3, or less than or equal to 0.7, reflecting a 30% difference compared to the entire sample.

Supplementary Table 3. Sensitivity analysis cox proportional hazard models associating study arm with last day of data transmission, censoring on patient death and adjusting for patient-level sociodemographic characteristics. Models were fit separately for each behavioral phenotype.

	Phenotype 1 n = 158, n events = 65		Phenotype 2 n = 105, events = 28 (26.7%)		Phenotype 3 n = 86, events = 31 (36.0%)		Phenotype 4 n = 93, events = 44 (47.3%)	
Variable	*HR (95% CI)*	*p-value*	*HR (95% CI)*	*p-value*	*HR (95% CI)*	*p-value*	*HR (95% CI)*	*p-value*
Study Arm								
Smartphone	Ref.		Ref.		Ref.		Ref.	
Wearable	1.03 (0.62 - 1.73)	0.900	1.42 (0.62 - 3.24)	0.402	3.83 (1.55 - 9.43)	0.004*	1.54 (0.78 - 3.05)	0.211
Age	1.01 (0.98 - 1.03)	0.635	0.98 (0.94 - 1.03)	0.414	1.01 (0.97 - 1.05)	0.624	1.01 (0.98 - 1.05)	0.372
Gender								
Male	Ref.		Ref.		Ref.		Ref.	
Female	1.20 (0.66 - 2.17)	0.558	1.60 (0.66 - 3.85)	0.297	1.24 (0.53 - 2.90)	0.612	1.22 (0.56 - 2.66)	0.614
Race								
Hispanic	Ref.		Ref.		Ref.		Ref.	
Non-Hispanic Black	1.16 (0.36 - 3.70)	0.802	0.32 (0.05 - 2.17)	0.243	1.72 (0.17 - 17.40)	0.646	0.90 (0.17 - 4.58)	0.894
Non-Hispanic White	0.56 (0.16 - 1.98)	0.369	0.70 (0.15 - 3.21)	0.651	0.92 (0.09 - 9.28)	0.945	2.06 (0.43 - 9.77)	0.363
Other	1.34 (0.33 - 5.51)	0.681	0.38 (0.03 - 4.96)	0.464	0.68 (0.04 - 12.57)	0.797	2.79 (0.34 - 22.81)	0.338
Insurance Type								
Commercial	Ref.		Ref.		Ref.		Ref.	
Medicare	0.84 (0.44 - 1.60)	0.586	1.16 (0.40 - 3.37)	0.781	4.13 (1.36 - 12.57)	0.013*	0.97 (0.43 - 2.20)	0.947
Medicaid	1.25 (0.64 - 2.45)	0.505	13.41 (1.10 - 163.18)	0.042*	5.34 (1.54 - 18.57)	0.008*	2.04 (0.72 - 5.80)	0.179
Education Level								
Less than High School	Ref.		Ref.		Ref.		Ref.	
High School Graduate	0.72 (0.33 - 1.59)	0.421	0.31 (0.03 - 2.94)	0.310	4.03 (0.79 - 20.64)	0.094	1.59 (0.36 - 7.16)	0.543
College Graduate	0.57 (0.23 - 1.40)	0.221	0.22 (0.02 - 2.00)	0.177	7.47 (0.99 - 56.08)	0.051	0.84 (0.16 - 4.47)	0.842
Marital Status								
Single, never married	Ref.		Ref.		Ref.		Ref.	
Married or domestic partnership	0.92 (0.47 - 1.78)	0.803	1.80 (0.39 - 8.32)	0.454	0.53 (0.17 - 1.62)	0.265	1.06 (0.45 - 2.48)	0.893
Other	1.66 (0.77 - 3.56)	0.198	2.33 (0.40 - 13.69)	0.348	0.52 (0.13 - 2.12)	0.365	1.02 (0.37 - 2.79)	0.973
Household Income								
< 50,000	Ref.		Ref.		Ref.		Ref.	
50,000 - 100,000	0.74 (0.31 - 1.78)	0.504	0.54 (0.12 - 2.53)	0.434	1.65 (0.52 - 5.26)	0.394	0.67 (0.21 - 2.09)	0.491
>100,000	2.31 (0.63 - 8.40)	0.205	1.28 (0.24 - 6.99)	0.772	3.21 (0.66 - 15.71)	0.149	0.29 (0.08 - 1.08)	0.065
Declined to Respond	1.08 (0.62 - 1.87)	0.789	1.05 (0.23 - 4.86)	0.950	1.10 (0.40 - 3.03)	0.851	0.68 (0.31 - 1.49)	0.331
BMI	0.99 (0.96 - 1.02)	0.421	1.02 (0.96 - 1.08)	0.503	0.95 (0.91 - 1.00)	0.034*	1.02 (0.98 - 1.06)	0.290
CCI	0.98 (0.89 - 1.09)	0.755	1.22 (1.05 - 1.42)	0.009*	1.01 (0.88 - 1.15)	0.927	0.95 (0.83 - 1.09)	0.448

Abbreviations: HR, hazard ratio. CI, confidence interval. BMI, body mass index. CCI, charlson comorbidity index. * p-value is significant ($p < 0.05$)

Supplementary Table 4. ANOVA tests comparing proportion of data transmitted between device types, separately for each behavioral phenotype

		Smartphone *mean (SD)*	Wearable *mean (SD)*	*p-value*
Phenotype 1, n		82	76	
	Main Analysis (steps > 0)	0.65 (0.37)	0.58 (0.36)	0.246
	Sensitivity Analysis (steps > 1000)	0.36 (0.31)	0.44 (0.34)	0.156
Phenotype 2, n		53	52	
	Main Analysis (steps > 0)	0.76 (0.36)	0.74 (0.36)	0.774
	Sensitivity Analysis (steps > 1000)	0.54 (0.34)	0.57 (0.35)	0.643
Phenotype 3, n		53	33	
	Main Analysis (steps > 0)	0.78 (0.31)	0.59 (0.39)	0.014*
	Sensitivity Analysis (steps > 1000)	0.56 (0.32)	0.42 (0.36)	0.063
Phenotype 4, n		35	58	
	Main Analysis (steps > 0)	0.67 (0.41)	0.49 (0.41)	0.047*
	Sensitivity Analysis (steps > 1000)	0.42 (0.30)	0.36 (0.36)	0.383

* p-value is significant ($p < 0.05$)

Supplementary Table 5. Sensitivity analysis cox proportional hazard models associating behavioral phenotype with last day of data transmission, censoring on patient death and adjusting for patient-level sociodemographic characteristics. Models were fit separately for each study arm.

	Smartphone<br>n = 223, n events = 72 (32.3%)		Wearable<br>n = 219, n events = 96 (43.8%)	
Variable	*HR (95% CI)*	*p-value*	*HR (95% CI)*	*p-value*
Behavioral Phenotype				
Phenotype 1	1.06 (0.54 - 2.08)	0.865	0.54 (0.32 - 0.92)	0.024*
Phenotype 2	0.68 (0.29 - 1.56)	0.358	0.56 (0.29 - 1.07)	0.079
Phenotype 3	0.81 (0.39 - 1.71)	0.585	0.70 (0.36 - 1.34)	0.278
Phenotype 4	Ref.		Ref.	
Age	1.01 (0.99 - 1.04)	0.290	0.99 (0.97 - 1.02)	0.565
Gender				
Male	Ref.		Ref.	
Female	1.38 (0.82 - 2.31)	0.228	1.17 (0.73 - 1.85)	0.515
Race				
Hispanic	Ref.		Ref.	
Non-Hispanic Black	1.22 (0.38 - 3.94)	0.744	0.52 (0.21 - 1.30)	0.160
Non-Hispanic White	1.07 (0.34 - 3.39)	0.912	0.58 (0.23 - 1.46)	0.245
Other	0.97 (0.22 - 4.34)	0.973	0.67 (0.20 - 2.20)	0.509
Insurance Type				
Commercial	Ref.		Ref.	
Medicare	0.88 (0.51 - 1.53)	0.658	1.24 (0.70 - 2.19)	0.462
Medicaid	1.46 (0.73 - 2.90)	0.287	1.92 (1.00 - 3.72)	0.052
Education Level				
Less than High School	Ref.		Ref.	
High School Graduate	1.92 (0.64 - 5.79)	0.247	0.65 (0.33 - 1.31)	0.227
College Graduate	1.00 (0.30 - 3.34)	0.999	0.71 (0.33 - 1.53)	0.377
Marital Status				
Single, never married	Ref.		Ref.	
Married or domestic partnership	0.88 (0.47 - 1.66)	0.699	0.95 (0.54 - 1.67)	0.849
Other	1.06 (0.51 - 2.21)	0.879	1.18 (0.62 - 2.24)	0.609
Household Income				
< 50,000	Ref.		Ref.	
50,000 - 100,000	1.27 (0.61 - 2.66)	0.529	0.62 (0.30 - 1.27)	0.190
>100,000	1.55 (0.63 - 3.83)	0.338	0.64 (0.28 - 1.46)	0.289
Declined to Respond	1.45 (0.81 - 2.60)	0.212	0.75 (0.45 - 1.24)	0.255
BMI	0.97 (0.94 - 1.00)	0.038*	1.01 (0.98 - 1.03)	0.646
CCI	1.06 (0.97 - 0.15)	0.190	1.02 (0.94 - 1.10)	0.662

Abbreviations: HR, hazard ratio. CI, confidence interval. BMI, body mass index. CCI, Charlson Comorbidity Index.

* p-value is significant ($p < 0.05$)

Supplementary Table 6. ANOVA tests comparing proportion of data transmitted between behavioral phenotypes, separately by device type

	Phenotype 1 *mean (SD)*	Phenotype 2 *mean (SD)*	Phenotype 3 *mean (SD)*	Phenotype 4 *mean (SD)*	*p-value*
Smartphone, n	82	53	53	35	
Main Analysis (steps > 0)	0.65 (0.37)	0.76 (0.36)	0.78 (0.31)	0.67 (0.41)	0.301
Sensitivity Analysis (steps > 1000)	0.36 (0.31)	0.54 (0.34)	0.56 (0.32)	0.42 (0.30)	0.057
Wearable, n	76	52	33	58	
Main Analysis (steps > 0)	0.58 (0.36)	0.74 (0.36)	0.59 (0.39)	0.49 (0.41)	0.116
Sensitivity Analysis (steps > 1000)	0.44 (0.34)	0.57 (0.35)	0.42 (0.36)	0.36 (0.36)	0.093

* p-value is significant ($p < 0.05$)
